# Cancer Fields

**Published:** 1899-04-22

**Authors:** 


### CANCER FIELDS.

The Editor of the *Practitioner* has done good service in collecting together a series of papers treating of many of the most vexed questions which are now current in regard to cancer. The many difficulties which are inherent in the statistical study of disease are well illustrated by the fact that doubt still hovers over questions as to the reality of the increase of cancer which statistics seem to show; as to the meaning of the facts which have been observed in regard to its geographical distribution; and as to the teaching to be derived from the very curious and interesting observations which have been made from time to time in regard to the undue liability of certain houses to become the homes of cancer. Such problems as these might well be put on one side for the present were it not that, as things stand just now, the most solid observations tend to indicate that cancer may possibly be a parasitic disease, and thus anything which shows a similarity in general behaviour between cancer and those diseases whose parasitic origin is already generally accepted is of much value as a confirmation of the hypothesis, although in itself it may be quite insufficient to prove what it so strongly suggests. This, we take it, is the main value of such observations as those that have been made by Dr. Alfred Havilland on the geographical distribution of cancer, and the very remarkable investigations by Mr. D'Arcy Power in regard to its local distribution and on "cancer houses." According to the former the districts having the highest death-rates from cancer, among females, are invariably associated with seasonally-flooded areas traversed by or in close propinquity to fully-formed rivers; while those having the lowest death-rates from cancer are situated on elevated land where the drainage is good and the physical features such as to preclude the possibility of floods—areas whence rivers derive their sources, and where, in fact, they are not fully formed; and, after carefully going over the added statistics of later periods, he still finds the same thing, and says: "Thus we find ourselves asking the same question that rose to the lips . . . . in November, 1868, 'How is it that limestones are always associated in England and Wales with the lowest mortality form from cancer, and flooded clays with the highest?' This question still remains unanswered."

